# The protein structure determines the sensitizing capacity of Brazil nut 2S albumin (*Ber e1*) in a rat food allergy model

**DOI:** 10.1186/2045-7022-3-36

**Published:** 2013-11-04

**Authors:** Jolanda HM Van Bilsen, Léon MJ Knippels, André H Penninks, Willem F Nieuwenhuizen, Harmen HJ De Jongh, Stef J Koppelman

**Affiliations:** 1 TNO, Zeist, Netherlands; 2 Nutricia Research, Utrecht, Netherlands; 3 Gilead Sciences Netherlands B.V, Amsterdam, Netherlands; 4 TI Food and Nutrition, Wageningen, Netherlands; 5 Food Allergy Research and Resource Program, University of Nebraska, Lincoln, Nebraska, USA

**Keywords:** Brazil nut 2S albumin, Ber e1, Food allergen, Rats, Reduction, Alkylation, Allergenicity, Immunogenicity

## Abstract

It is not exactly known why certain food proteins are more likely to sensitize. One of the characteristics of most food allergens is that they are stable to the acidic and proteolytic conditions in the digestive tract. This property is thought to be a risk factor in allergic sensitization. The purpose of the present study was to investigate the contribution of the protein structure of 2S albumin (*Ber e1*), a major allergen from Brazil nut, on the sensitizing capacity *in vivo* using an oral Brown Norway rat food allergy model. Disulphide bridges of 2S albumin were reduced and alkylated resulting in loss of protein structure and an increased pepsin digestibility *in vitro*. Both native 2S albumin and reduced/alkylated 2S albumin were administered by daily gavage dosing (0.1 and 1 mg) to Brown Norway rats for 42 days. Intraperitoneal administration was used as a positive control. Sera were analysed by ELISA and passive cutaneous anaphylaxis. Oral exposure to native or reduced/alkylated 2S albumin resulted in specific IgG1 and IgG2a responses whereas only native 2S albumin induced specific IgE in this model, which was confirmed by passive cutaneous anaphylaxis. This study has shown that the disruption of the protein structure of Brazil nut 2S albumin decreased the sensitizing potential in a Brown Norway rat food allergy model, whereas the immunogenicity of 2S albumin remained preserved. This observation may open possibilities for developing immunotherapy for Brazil nut allergy.

## Introduction

Humans rather frequently suffer from allergic reactions after consumption of dietary proteins. The prevalence of food allergy is approximately 1-2% in adults and 6-8% in children and most food allergies are mediated by antigen-specific IgE and are characterized as type-I reactions [1]. Cases of severe allergic reactions including anaphylaxis were reported for Brazil nut (*Bertholletia excelsa*) [2] and the storage protein 2S albumin was identified as allergen and classified as *Ber e1*. It has gained particular interest as in the early 1990’s it was considered to transfer the gene coding for the Brazil nut 2S albumin by transgenic techniques to soybean in order to improve its nutritional value for animal feed which however never reached the market due to the allergenic nature of the Brazil nut 2S albumin [3].

An important characteristic of food allergens is that they tend to be more stable to the proteolytic and acidic conditions of the digestive tract, which results in an increased probability of reaching the intestinal mucosa, where absorption can occur and allergic sensitization may be induced [4,5]. Many of the major food allergens are comparatively resistant to digestion, and this general characteristic has been considered a risk factor for food allergy induction. Furthermore, *in vitro* digested Brazil nut 2S albumin retains about one quarter of its IgE-binding potency [6], and similar observations were made for 2S albumin-like allergens from peanut [7]. This suggests that even if 2S albumins are digested, their allergenicity in terms of IgE-binding is not completely abolished. Taken together, resistance to digestion does not provide sufficient information for safety aspects in terms of risk of allergenicity.

The purpose of this study is to evaluate the relation between specific structural aspects of Brazil nut 2S albumin and its sensitizing capacity. Therefore, we prepared two forms of 2S albumin from Brazil nuts, a native- and a reduced/alkylated 2S albumin (2S albumin-R) in which the disulphide bridges are reduced. Both have the same amino acid sequence that provides the same set of potential antigenic linear epitopes, but with different structural organization. Reduction of the disulphide bridges of Brazil nut 2S albumin led to a remarkable decrease in stability toward digestion [8]. Both preparations are here tested in their ability to induce allergic sensitization in a Brown Norway rat model for food allergy.

## Findings

### Materials and methods

Disulphide bridges of 2S albumin were reduced and alkylated resulting in unfolding of the protein [8], here referred to as 2S albumin-R. To study the sensitizing capacity of the structurally different forms of 2S albumin [8]*in vivo* we used an oral Brown Norway rat food allergy model. Young male Brown Norway (BN) rats (3-4 weeks old at arrival) obtained from Charles River (Sulzfeld, Germany) were bred and raised on a commercially available Brazil nut free rodent diet (SDS Special Diet Service, LAD1 (E) SQC, Witham, England). For the oral sensitization animals (n = 10) were exposed by gavage dosing during 6 weeks to either 2S albumin-R or native 2S albumin (0.1 mg or 1 mg protein/ml tap water; 1 ml/animal) without the use of an adjuvant or only water (controls). Blood samples were obtained days 0, 28 and 42 and sera were prepared. Positive control animals (n = 5) were injected intraperitoneally (i.p.) with 0.5 ml of a 0.2 mg/ml RA- or native 2S albumin solution in sterile saline on days 0, 2, 4, 7, 9 and 11. In the positive control animals, the immune response was potentiated at day 0 with 0.2 ml of a 25 mg/ml Al(OH)3 adjuvant suspension in sterile saline mixed with the 0.5 ml of 2S albumin or 2S albumin-R solution. The animals were bled on day 28. The sera were analyzed for anti-native- and 2S albumin-R-specific IgG1 (Thelper-1 mediated), IgG2a and IgE (Thelper-2 mediated) titers by ELISA and by Passive Cutaneous Anaphylaxis (PCA) essentially as described previously [9]. Data were analysed using one-way ANOVA with Bonferroni’s post-hoc test using GraphPad Prism version 5.00 (GraphPad Software, San Diego, CA, USA). The welfare of the animals was maintained in accordance with the general principles governing the use of animals in experiments of the European Communities (Directive 86/209/EEC) and Dutch legislation (The experiments on Animals Act, 1997), which includes approval of the study by TNO's ethical review committee under DEC number 1732.

### Results and discussion

**Figure 1:**
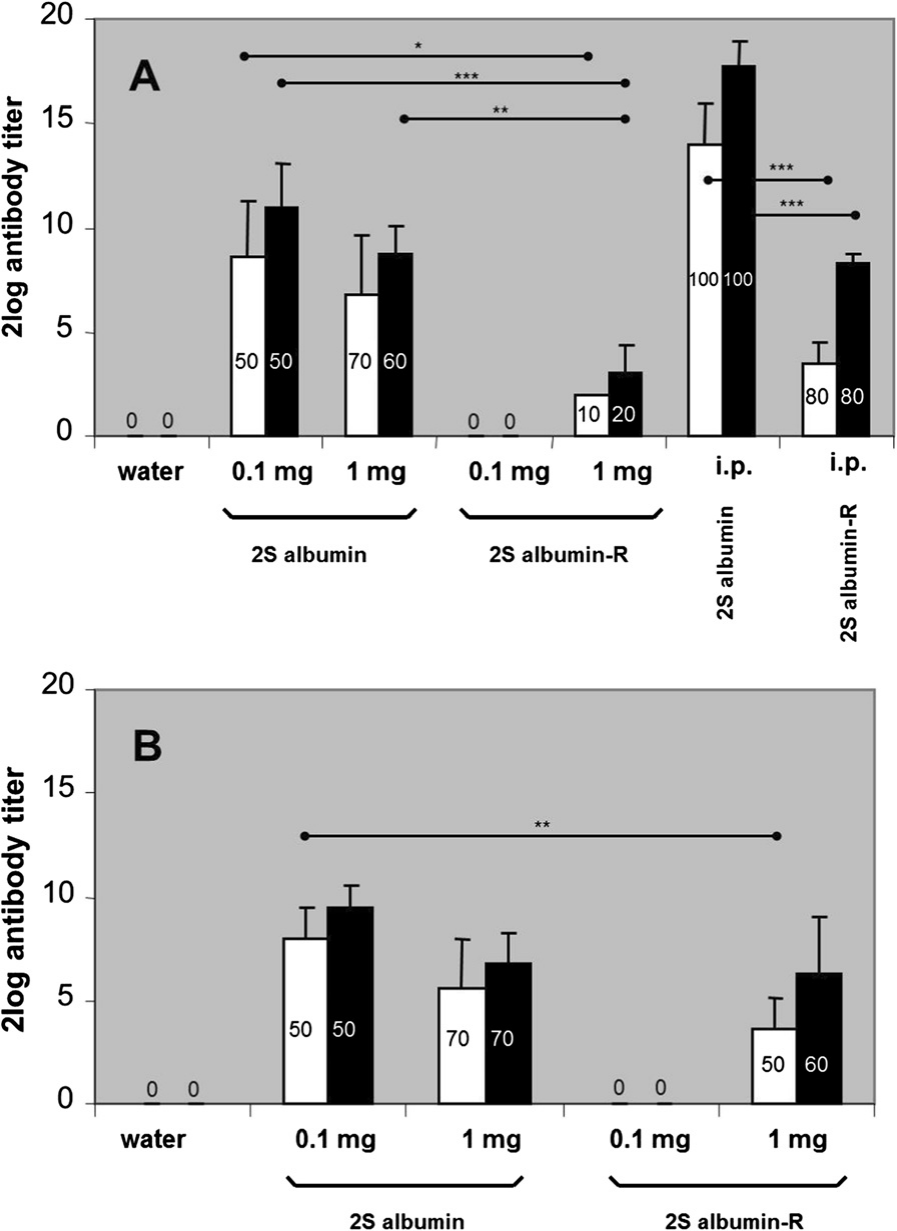
**2S albumin and 2S albumin-R-specific IgG1 (■) and IgG2a (□) titers were analyzed upon daily intra-gastric dosing of BN rats (n=10/group) with water, 0.1 mg and 1 mg 2S albumin or 2S albumin-R per rat for 28 days (A) or 42 days (B).** Positive control animals (n=5/group) received multiple i.p. sensitizations prior serum analyses at day 28 **(A)**. The data are presented as mean 2log Ig titer ± SD of the number of responding rats (indicated as percentage in the bars) per group. Statistical differences between oral dosing groups or between i.p. dosing groups are depicted (*p < 0.05, **p < 0.01, ***p < 0.001).

**Figure 2:**
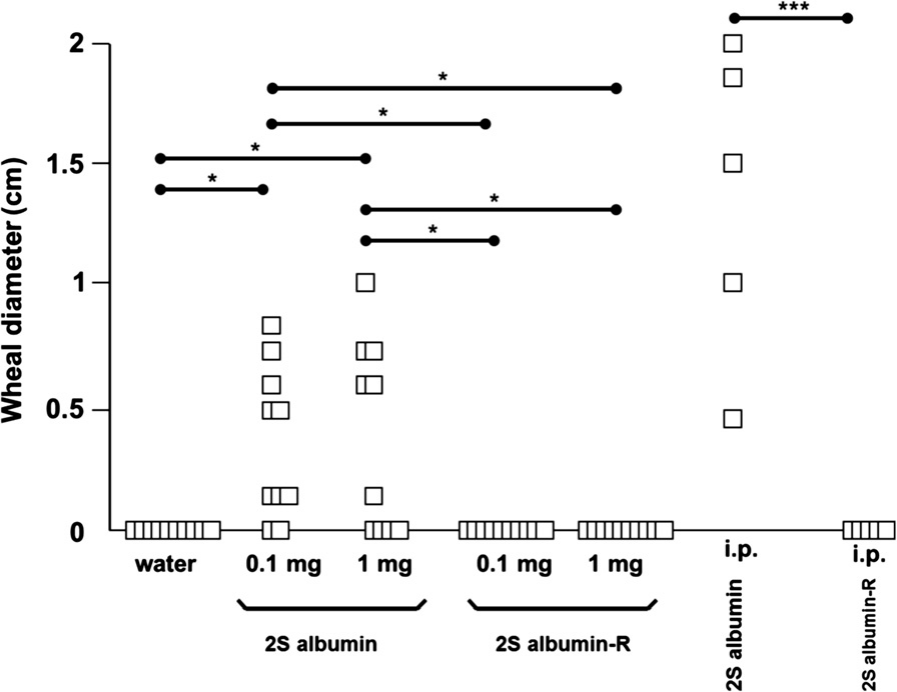
**IgE antibody responses as measured by PCA upon intra-gastric or i.p. sensitization.** 2S albumin and 2S albumin-R-specific IgE as measured by PCA tests were determined upon daily intragastric dosing of BN rats (n = 10) with water, 0.1 mg and 1 mg 2S albumin or 2S albumin-R per rat for 42 days or after i.p. sensitization (n = 5; day 28 sera), respectively. Data are given as the individual measured wheal diameter in cm (□) for each rat. Statistical differences between oral dosing groups or between i.p. dosing groups are depicted (**p < 05, ***p < 0.001).

From the animals orally exposed to native 2S albumin the majority developed 2S albumin-specific IgG1 and IgG2a responses at days 28 and 42 in a dose-dependent way whereas oral exposure to 2S albumin-R resulted in a lower number of responding animals (Figure 1A + B). At day 28, i.p. exposure of the animals to native 2S albumin resulted in the development of very pronounced 2S albumin-specific IgG1 and IgG2a responses in all animals (Figure 1A). I.p. exposure to 2S albumin-R resulted in 2S albumin-R-specific IgG1 and IgG2a responses in 80% of the animals. Both oral (0.1 and 1 mg protein/rat/day) and i.p. exposure of the animals to 2S albumin-R did not result in the development of 2S albumin-R-specific IgE antibodies. In contrast, oral dosing of the animals to either 0.1 mg or 1 mg native 2S albumin resulted in 2S albumin-specific IgE responses in 50% of the animals and i.p. exposure lead to 100% responders. These results were confirmed by PCA testing (Figure 2). These results show that reduction of disulfide bonds and concomittal loss of protein structure and an increased sensitivity for digestion of Brazil nut 2S albumin [8], decreases the prominent sensitizing potential of 2S albumin in the oral BN rat food allergy model. Not all animals developed specific antibody responses upon oral exposure to 2S albumin. This phenomenon is also observed with other food allergens like ovalbumin and cow’s milk using the described BN rat food allergy model [9]. While the allergenicity of 2S albumin-R was dramatically decreased, the immunogenicity of the protein still existed since specific IgG1 and IgG2a antibodies against 2S albumin were produced, although at a lower level compared to native 2S albumin.

Evidence for an important role of the structural conformation (the secondary and tertiary structure) of the protein with respect to food protein allergenicity also comes from animal studies. Prefeeding of an endopeptidase inhibitor (aprotinin) to mice results in an inhibition of oral tolerance induction by protein feeding [10] while feeding of protein antigen to mice is known to induce substantial systemic tolerance for specific antibody and cell mediated immune responses under normal circumstances [11]. Recently it was shown that by regulating the gastric pH the dose-dependency of food allergy induction was influenced [12]. The effect of anti-ulcer drugs that increase the gastric pH on the formation of IgE in humans was investigated previously [13]. It was found that pre-existing IgE was boosted to higher levels and that *de novo* IgE towards digestion-labile food proteins was induced. The implications of regulating the gastric pH on the risks for food allergic consumers were recently reviewed [14]. While the above mentioned studies investigated the effect of decreasing the susceptibility to proteolysis, we investigated the effect of increasing the susceptibility to proteolysis.

Disruption of the structural conformation of protein by alkylation/denaturation has previously been used to generate hypoallergenic variants of Ara h2 [15] and Pru p3 [16], respectively the major peanut and peach allergen. Although no hypoallergenic derivatives have received marketing authorization to date, they are suggested to be promising vaccine candidates for immunotherapy. The reduced IgE-antibody-binding capacity reduces the frequency of adverse reactions during allergen-specific immunotherapy, allowing the application of higher amounts of allergen with a reduced risk of serious adverse effects [17,18]. Moreover, T cell immunogenicity needs to be preserved to maintain the therapeutic potential [19,20]. In line with this, we here show that the reduced/alkylated Brazil nut 2S albumin showed a reduction in biologically active IgE (by PCA), reflecting a reduced allergenic potential, whereas the immunogenicity remained intact.

Interestingly, if the native 2S albumin and 2S albumin-R form were injected i.p. together with alum as an adjuvant, specific IgE antibodies were only observed in animals treated with the native 2S albumin. As upon i.p. exposure degradation by the digestive tract will be absent and moreover the tolerogenic mucosal sites are bypassed, it is suggested that other aspects may also influence the potential allergenicity of a protein. It is known that proteases present in dendritic cells (DC) generate peptides from foreign and self proteins for eventual capture and display to T cells, demonstrating that individual proteolytic enzymes may have a clear contribution to antigen processing [21]. In fact, it was recently shown that the immunogenicity of Bet v1, the major birch pollen allergen, could be influenced by changing the susceptibility towards digestion [22]. Similar observations were made for 2S albumins from other allergenic plant foods when injected i.p. [16]. Native 2S albumin compared to 2S albumine-R is relatively stable to digestion [8], an altered degradation by individual proteolytic enzymes and subsequent presentation of both peptide fragments by antigen presenting cells may thus lead to an altered antibody response. This may explain the observed difference in response against 2S albumin and 2S albumin-R upon i.p. administration. In conclusion, the results of this study demonstrate the importance of the protein structure of Brazil nut 2S albumin in inducing food allergy. The observation that modified Brazil nut 2S albumin is still immunogenic while its allergenicity is reduced opens possibilities for developing immunotherapy for Brazil nut allergy.

## Abbreviations

2S albumin-R: Reduced and alkylated 2S albumin; BN: Brown Norway; DC: Dendritic cells; i.p: Intraperitoneally; PCA: Passive cutaneous anaphylaxis.

## Competing interests

The authors report no conflicts of interest. SJK is consultant to DBV Technologies, a company involved in developing epicutaneous immunotherapy for peanut allergy.

## Authors’ contributions

JHMB carried out data analysis. LMJK carried out animal data acquisition and analysis. WFN and HHJJ prepared the proteins. AHP participated in the coordination of the animal studies. SJK was responsible for overall design of the studies and the qualification of the proteins. All authors were involved in the preparation of the manuscript and gave their final approval of the submitted version.
